# Loss of C/EBPδ Exacerbates Radiation-Induced Cognitive Decline in Aged Mice due to Impaired Oxidative Stress Response

**DOI:** 10.3390/ijms20040885

**Published:** 2019-02-18

**Authors:** Sudip Banerjee, Tyler Alexander, Debajyoti Majumdar, Thomas Groves, Frederico Kiffer, Jing Wang, Akshita Gorantla, Antiño R. Allen, Snehalata A. Pawar

**Affiliations:** Division of Radiation Health, Department of Pharmaceutical Sciences, College of Pharmacy, University of Arkansas for Medical Sciences, Little Rock, AR 72205, USA; SBanerjee@uams.edu (S.B.); Tyler.alexander@stjude.org (T.A.); DMajumdar@uams.edu (D.M.); tuk52207@temple.edu (T.G.); FCKiffer@uams.edu (F.K.); JWang2@uams.edu (J.W.); akshitagorantla@gmail.com (A.G.)

**Keywords:** Cebpd, C/EBPδ, ionizing radiation, hippocampus, behavior, novel object recognition, spatial learning, short-term memory, oxidative stress, reactive oxygen species

## Abstract

Aging is characterized by increased inflammation and deterioration of the cellular stress responses such as the oxidant/antioxidant equilibrium, DNA damage repair fidelity, and telomeric attrition. All these factors contribute to the increased radiation sensitivity in the elderly as shown by epidemiological studies of the Japanese atomic bomb survivors. There is a global increase in the aging population, who may be at increased risk of exposure to ionizing radiation (IR) as part of cancer therapy or accidental exposure. Therefore, it is critical to delineate the factors that exacerbate age-related radiation sensitivity and neurocognitive decline. The transcription factor CCAAT enhancer binding protein delta (C/EBPδ) is implicated with regulatory roles in neuroinflammation, learning, and memory, however its role in IR-induced neurocognitive decline and aging is not known. The purpose of this study was to delineate the role of C/EBPδ in IR-induced neurocognitive decline in aged mice. We report that aged *Cebpd*^−/−^ mice exposed to acute IR exposure display impairment in short-term memory and spatial memory that correlated with significant alterations in the morphology of neurons in the dentate gyrus (DG) and CA1 apical and basal regions. There were no significant changes in the expression of inflammatory markers. However, the expression of superoxide dismutase 2 (SOD2) and catalase (CAT) were altered post-IR in the hippocampus of aged *Cebpd*^−/−^ mice. These results suggest that *Cebpd* may protect from IR-induced neurocognitive dysfunction by suppressing oxidative stress in aged mice.

## 1. Introduction

There is strong evidence for multifaceted damage to the brain after IR exposure provided by epidemiological studies on the atomic bomb survivors, cancer survivors, and occupational cohorts [1]. The advent of modern medicine has led to a substantial increase in human lifespan and the number of older people among the global population is currently higher and still expanding. The hallmarks of aging are characterized by increased inflammation and deterioration of the cellular stress responses such as the oxidant/antioxidant equilibrium, DNA damage repair fidelity, and telomeric attrition [2]. All these factors contribute to the increased radiation sensitivity in the elderly [2]. Therefore, understanding the mechanistic processes involved in age-related radiation sensitivity is of utmost relevance, particularly in view of the increasing aging population who may be exposed to IR as part of cancer therapy or accidental exposure to IR. However, very little is known about the molecular mechanism of increased sensitivity to radiation during aging.

Exposure to IR leads to the expression of pro-inflammatory cytokines and reactive oxygen species (ROS) in the brain areas [3,4,5,6,7]. Loss of verbal memory, spatial memory, attention, and novel problem-solving ability are the hallmarks of radiation-induced cognitive impairment [8,9,10,11,12,13]. A major role in learning, consolidation, and retrieval of information is done by the hippocampus [13,14]. Hippocampus, the main region of the brain where neurogenesis occurs throughout one’s lifetime, is highly susceptible to radiation-induced damage [15]. An important player in neurogenesis is the identification of mitochondrial function and, together with observations that mitochondria are targets for ionizing radiation effects, potentially implied mitochondrial dysfunction in radiation-induced deficit of hippocampal neurogenesis-dependent cognition [4,15,16]. Regulation of adult neurogenesis depends on the metabolic status of the animal [17]. Previous studies have reported that exposure to total body irradiation (TBI) induces acute alterations in neuronal structure and early cognitive changes [18,19]. Data from us and others show that doses of high linear energy transfer radiation from 0.1–1 Gy cause significant, dose-responsive reductions in hippocampal dendritic complexity and spine density, which last for up to nine months post-irradiation [20,21,22,23]. Therefore, the purpose of this study was to investigate the early effects of TBI on neurocognitive functions in *Cebpd*^+/+^ and *Cebpd^−/−^* mice. In susceptibility to radiation, age also plays a major role [2,15,24]. Although much is known about the late effects of radiation on neurocognitive deficits, very little is known about the molecular markers and morphological changes that occur in the brain in response to acute effects of IR exposure on neurocognitive functions in the context of aging. Understanding these molecular mechanisms of IR’s early effects on neurocognitive dysfunction would enable the development of novel interventions to alleviate the adverse effects due to accidental exposure or as part of radiotherapy to the brain.

The transcription factor CCAAT enhancer binding protein delta (C/EBPδ) is implicated in having a regulatory role in diverse biological functions such as acute-phase response (reaction to inflammation), growth arrest, apoptosis, differentiation, stem cell self-renewal, and tumor suppression [25]. Work by us and others has shown a role for C/EBPδ in maintaining genomic stability, cell cycle arrest, DNA damage repair, and oxidative stress [26,27,28,29,30,31]. There are several compelling studies that point to a role of C/EBPδ in neuroinflammation in diseases such as Alzheimer’s, where the ablation of C/EBPδ is shown to confer a protective role [32,33]. We have previously shown a role for C/EBPδ in radiation response, in promoting post-radiation survival by protection against radiation-induced hematopoietic and intestinal injury, and in modulating basal as well as IR-induced oxidative stress and mitochondrial dysfunction [34,35]. In the present study, we investigated whether the loss of *Cebpd* exacerbates radiation-induced cognitive deficits due to an impaired ability to detoxify IR-induced ROS and/or inflammation.

## 2. Results

### 2.1. Irradiation Impairs Short-Term Memory during Y-Maze Test in Cebpd^−/−^ Mice

**Figure 1:**
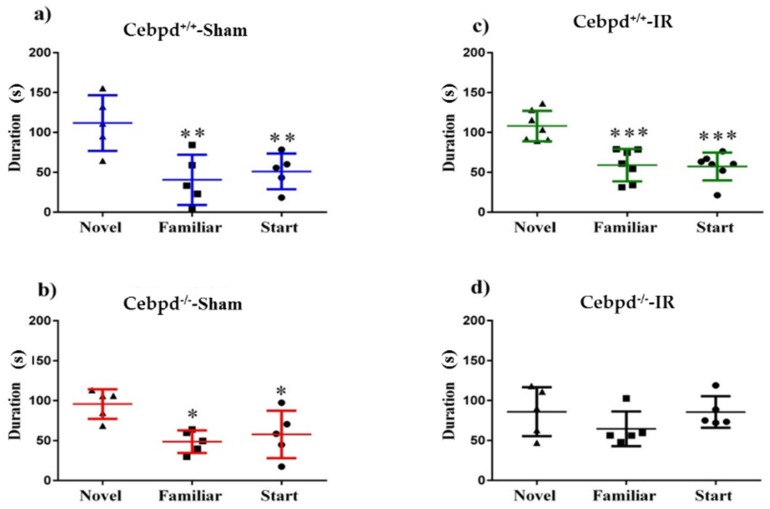
Short-term memory analyzed by Y-maze test in sham and irradiated aged *Cebpd*^+/+^ and *Cebpd*^−/−^ mice. (**A**–**C**) *Cebpd*^+/+^-sham, *Cebpd*^+/+^-IR, and *Cebpd*^−/−^-sham mice were able to successfully distinguish the novel arm, by spending significantly more time exploring it. (**D**) *Cebpd*^−/−^-IR mice were not able to distinguish between the three Y-maze arms, and spent an approximately equal time exploring all arms failing to recognize the novel environment when exposed to it 4 h later. N = 5/7 mice/treatment, Average ± SEM; * *p* < 0.05, ** *p* < 0.01, *** *p* < 0.001.

The Y-maze is an established behavioral assay for short-term spatial memory [36]. The amount of time a mouse spends exploring a novel arm relative to the familiar arm in the testing phase is indicative of its ability to retain the spatial memory encoded during familiarization. We observed that *Cebpd*^+/+^-sham, *Cebpd*^+/+^-IR, and *Cebpd*^−/−^-sham groups displayed significant differences in exploration between the maze arms during the testing phase (*Cebpd*^+/+^-sham: F _(2, 12)_ = 8.16, *p* < 0.01; *Cebpd*^+/+^-IR: F _(2, 18)_ = 16.21; *p* < 0.001; *Cebpd*^−/−^-sham: F _(2, 12)_ = 6.56; *p* < 0.05). Post-hoc tests indicate that *Cebpd*^+/+^-sham animals spent significantly more time exploring the novel than the familiar (*p* < 0.05) or start (*p* < 0.01; Figure 1A) arms. Similarly, the *Cebpd*^+/+^-IR treatment group also spent significantly more time exploring the novel than the familiar (*p* < 0.001) or start (*p* < 0.001; Figure 1B) arms. Next, the *Cebpd*^−/−^-sham group was also successful in exploring the novel arm for longer periods of time than the familiar or start arms (*p* < 0.05; Figure 1C). However, the *Cebpd*^−/−^-IR animals displayed impaired short-term memory (F _(2, 12)_ = 1.25; *p* = 0.32; Figure 1D) spending equal amounts of time exploring the novel and start arms.

### 2.2. Irradiation Impairs Spatial Memory in Aged Cebpd^−/−^ Mice

**Figure 2:**
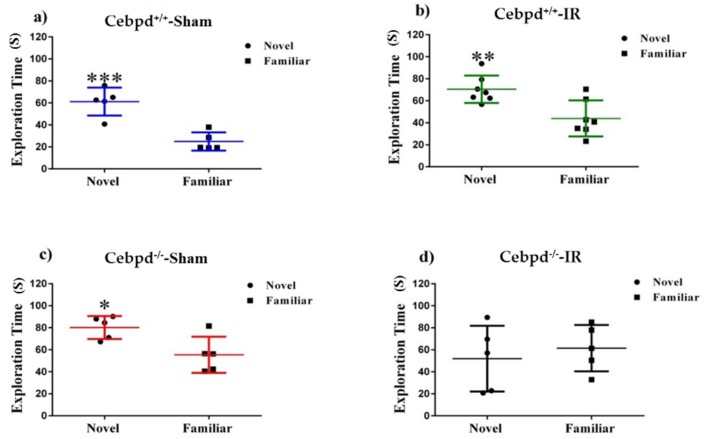
NOR of sham-irradiated and irradiated aged *Cebpd*^+/+^ and *Cebpd*^−/−^ mice. (**A**) *Cebpd*^+/+^-sham, (**B**) *Cebpd*^+/+^-IR, and (**C**) *Cebpd*^−/−^-sham irradiated mice showed novel object recognition and spent more time exploring the novel than the familiar object. However, *Cebpd*^−/−^-IR (**D**) mice did not show any preference for the novel object. N = 5/7 mice/treatment. Average ± SEM; * *p* < 0.05, ** *p* < 0.01, *** *p* < 0.001.

We used the novel object recognition (NOR) task to assess non-spatial declarative memory [37]. Rodents naturally orient their head toward novel stimuli, behavior that provides a simple and effective method for quantifying visual recognition [38]. Visuospatial orientation toward an object will attenuate with arena exposure time (habituation), and contrasting exploration of a novel versus a familiar object provides an index of object recognition and discrimination. Habituation learning occurs when animals’ response to a stimulus lowers with increased exposure. Locomotor activity was tracked on the two empty arena habituation days, and the difference between total distances moved between open arena days 1 and 2 serve as a metric for habituation learning. There was no significant difference in distance moved day 1 (F _(3, 18)_ = 0.73; *p* = 0.55) nor day 2 (F _(3, 18)_ = 1.79; *p* = 0.18). During familiarization (day 3), mice were placed in the open field box with two identical objects. On day 4, one of the objects (henceforth “familiar”) was replaced with a novel object. Statistical analysis of total object exploration in test sessions revealed that *Cebpd*^+/+^-sham (*t* = 5.33, *p* = 0.007; Figure 2A), *Cebpd*^+/+^-IR (*t* = 3.40, *p* = 0.005; Figure 2B) and *Cebpd*^−/−^-sham (*t* = 2.85, *p* = 0.02; Figure 2C) mice showed novel object recognition and visited the novel object significantly more than the familiar object. However, radiation exposure significantly impaired *Cebpd*^−/−^ mice (Figure 2D) as they did not show any preferences for the novel object. Discrimination ratios provide a basis for interpreting animals’ ability to remember or forget a novel arm or object. A positive ratio can be interpreted as animals successfully discriminating between two objects, and a negative ratio implies “forgetting” an object [39]. Radiation resulted in a negative discrimination ratio for *Cebpd*^−/−^-IR mice (F _(3, 17)_ = 4.42; *p* < 0.05; Figure S1, Supplementary Materials).

#### 2.2.1. Dendritic Morphology of Dentate Gyrus Granule Neurons is Significantly Altered in Irradiated Cebpd^−/−^ Mice

For morphological quantification of hippocampal neurons, we measured length and branching of the granule cells in the dentate gyrus (DG) and pyramidal neurons in the CA1 region from 5 *Cebpd*^−/−^ and 7 *Cebpd*^+/+^ mice. First, we examined dendritic complexity in the DG between treatment groups. An ANOVA found differences in dendritic complexity (F _(3, 12)_ = 9.81; *p* < 0.001). Multiple comparisons show a marked decrease in complexity between *Cebpd*^−/−^sham compared to *Cebpd*^−/−^-IR (*p* < 0.01; see Table 1). There were no significant differences between *Cebpd*^+/+^-sham compared to *Cebpd*^−/−^-sham nor between *Cebpd*^+/+^-sham compared to *Cebpd*^+/+^-IR. The variables that define dendritic complexity changed due to irradiation to similar extents as compared to sham. We observed decreases in dendritic length (F _(3, 12)_ = 11.78; *p* < 0.001) and total branch points (F _(3, 12)_ = 19.88; *p* < 0.0001; see Table 1).

**Figure 3:**
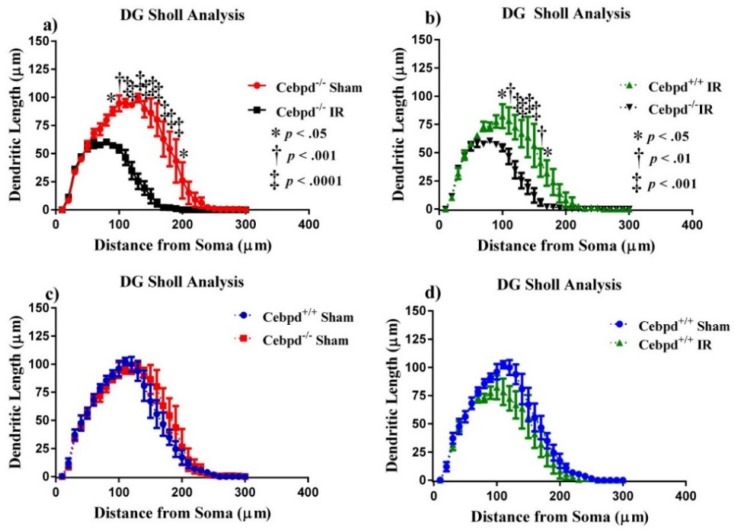
Sholl analyses of neurons in the dentate gyrus. (**A**) Dendritic length, measured by Sholl analysis, radiation greatly decreased length at 90–100 µm from the soma when *Cebpd*^−/−^-sham were compared to *Cebpd*^−/−^-IR. (**B**) Treatment decreased length at 100–170 µm from the soma when *Cebpd*^+/+^-IR were compared to *Cebpd*^−/−^-IR. There were no significant differences observed when (**C**) *Cebpd*^+/+^-sham were compared to *Cebpd*^−/−^-sham or (**D**) *Cebpd*^+/+^-sham were compared to *Cebpd*^+/+^-IR. Average ± SEM (*n* = 5); * *p* < 0.05, ^†^ *p* < 0.01. ^‡^ *p* < 0.001.

The effect of irradiation was found to be associated with a different distribution of dendritic branches over the entire tree in the DG, as determined by ANOVA. We detected significant interactions between treatment and dendritic length (F _(89,348)_ = 12.83; *p* < 0.0001). We also found significant main effects of Sholl dendritic length (F _(29,348)_ = 110.4; *p* < 0.0001) and main effect of treatment (F _(3, 12)_ = 11.78; *p* < 0.001). We next performed post-hoc analyses, which revealed a decrease in the dendritic length significantly evident when *Cebpd*^−/−^-sham were compared to *Cebpd*^−/−^-IR. Analysis revealed a significant decrease in dendritic length at 90–190 µm (Holm-Sidak’s multiple comparisons: 90 µm, *p* < 0.05; 100 µm, *p* < 0.001; 110–190 µm, *p* < 0.0001; Figure 3). We found no significant interactions between genotype and dendritic Sholl length (F _(29,174)_ = 0.81; *p* = 0.74; Figure 3) when *Cebpd*^+/+^-sham were compared to *Cebpd*^−/−^-sham. Nor was there a significant interaction between treatment and dendritic Sholl (F _(29,174)_ = 0.92; *p* = 0.57; Figure 3) when *Cebpd*^+/+^-sham were compared to *Cebpd*^+/+^-IR.

#### 2.2.2. Dendritic Morphology of CA1 Apical Neurons is Significantly Altered in Irradiated *Cebpd^−/−^* Mice

We next examined dendritic complexity in the CA1 neurons between treatment groups. An ANOVA found differences in dendritic complexity (F _(3, 12)_ = 6.50; *p* < 0.01). Multiple comparisons show a marked decrease in complexity between *Cebpd*^+/+^-sham compared to *Cebpd*^+/+^-IR (*p* < 0.05). We also observed decreases in dendritic length (F _(3, 12)_ = 9.06; *p* < 0.01) and total branch points (F _(3, 12)_ = 7.80; *p* < 0.01; see Table 2) in both dosage groups.

**Figure 4:**
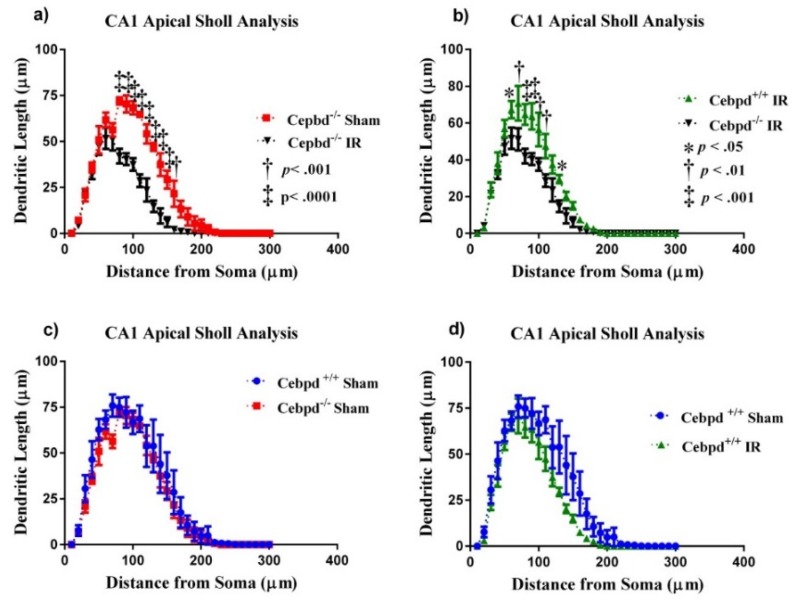
Sholl analyses of neurons in the CA1 apical. (**A**) Dendritic length, measured by Sholl analysis radiation decreased length at 80–160 µm from the soma when *Cebpd*^−/−^-sham were compared to *Cebpd*^−/−^-IR. (**B**) Treatment greatly decreased length at 80–160 µm from the soma when *Cebpd*^+/+^-IR were compared to *Cebpd*^−/−^ -IR. There were no significant differences observed when (**C**) *Cebpd*^+/+^-sham were compared to *Cebpd*^−/−^-sham or (**D**) *Cebpd*^+/+^-sham were compared to *Cebpd*^+/+^-IR. Average ± SEM (*n* = 5) * *p* < 0.05, ^†^ *p* < 0.01. ^‡^ *p* < 0.001.

We report significant interactions between treatment groups and dendritic Sholl length in the CA1 apical neurons. Similar to what was seen in the DG, we detected significant interactions between treatment and dendritic length (F _(87,348)_ = 2.54; *p* < 0.0001). We also found significant main effects of Sholl dendritic length (F _(29,348)_ = 127.1; *p* < 0.0001) and main effect of treatment (F _(3, 12)_ = 11.78; *p* < 0.001). We next performed post-hoc analyses, which revealed a decrease in the dendritic length significantly evident when *Cebpd*^−/−^-sham were compared to *Cebpd*^−/−^-IR. Analysis revealed a significant decrease in dendritic length at 80–160 µm (Holm-Sidak’s multiple comparisons: 80–150 µm, *p* < 0.0001; 160 µm, *p* < 0.001; Figure 4). We found no significant interactions between genotype and dendritic Sholl length (F _(29,174)_ = 0.41; *p* = 0.99; Figure 4) when *Cebpd*^+/+^-sham were compared to *Cebpd*^−/−^-sham. Nor was there a significant interaction between treatment and dendritic Sholl (F _(29,174)_ = 1.10; *p* = 0.34; Figure 4) when *Cebpd*^+/+^-sham were compared to *Cebpd*^+/+^-IR.

#### 2.2.3. Dendritic Morphology of CA1 Basal Neurons is Significantly Altered in Irradiated *Cebpd^−/−^* Mice

**Figure 5:**
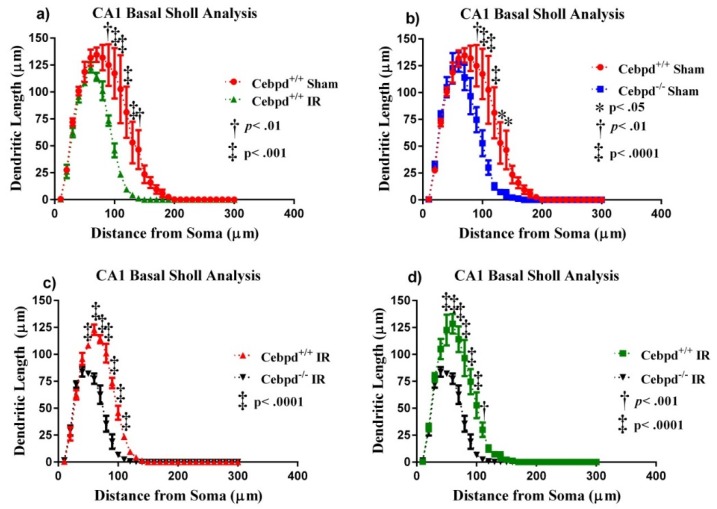
Sholl analyses of neurons in the CA1 basal. (**A**) Dendritic length, measured by Sholl analysis radiation decreased length at 90–140 µm from the soma when *Cebpd*^+/+^-sham were compared to *Cebpd*^+/+^-IR. (**B**) There was a decrease in length at 90–140 µm from the soma when *Cebpd*^+/+^-sham were compared to *Cebpd*^−/−^ -sham. (**C**) Radiation decreased length at 50–110 µm from the soma when *Cebpd*^+/+^-IR were compared to *Cebpd*^−/−^-IR. (**D**) Treatment greatly decreased length at 50–110 µm from the soma when *Cebpd*^−/−^-sham were compared to *Cebpd*^−/−^-IR. Average ± SEM (*n* = 5).

In the CA1 basal pyramidal dendrites, the ANOVA also found differences in dendritic complexity (F _(3, 12)_ = 6.35; *p* < 0.01). Multiple comparisons show a significant decrease in complexity when *Cebpd*^+/+^-IR were compared to *Cebpd*^−/−^-IR (*p* < 0.05). We also observed decreases in dendritic length (F _(3, 12)_ = 13.64; *p* < 0.001) and total branch points (F _(3, 12)_ = 8.80; *p* < 0.01; see Table 2). We detected significant interactions between treatment and dendritic length (F _(87,348)_ = 8.06; *p* < 0.0001). We also found significant main effects of Sholl dendritic length (F _(29,348)_ = 178.5; *p* < 0.0001) and main effect of treatment (F _(3, 12)_ = 12.31; *p* < 0.001). Post-hoc analyses revealed a significant decrease in the dendritic length when *Cebpd*^+/+^-sham were compared to *Cebpd*^+/+^-IR at 90–140 µm from the soma (Holm-Sidak’s multiple comparison: 90 µm, *p* < 0.01; 100–130 µm, *p* < 0.001; 140 µm, *p* < 0.01 Figure 5). When *Cebpd*^+/+^-sham were compared to *Cebpd*^−/−^-sham, analysis revealed a significant decrease in dendritic length at 90–130 µm (Holm-Sidak’s multiple comparisons: 90 µm, *p* < 0.01; 100–120 µm, *p* < 0.0001; 130–140 µm, *p* < 0.05; Figure 5). When *Cebpd*^−/−^-sham were compared to *Cebpd*^−/−^-IR, analysis revealed a significant decrease in dendritic length at 50–110 µm (Holm-Sidak’s multiple comparisons: 50–100 µm, *p* < 0.0001; 110 µm, *p* < 0.001; Figure 5).

### 2.3. Irradiated Cebpd^−/−^ Mice Show Impaired Expression of Antioxidant Response Proteins, but no Change in the Expression of Inflammatory Markers in the Hippocampus

**Figure 6:**
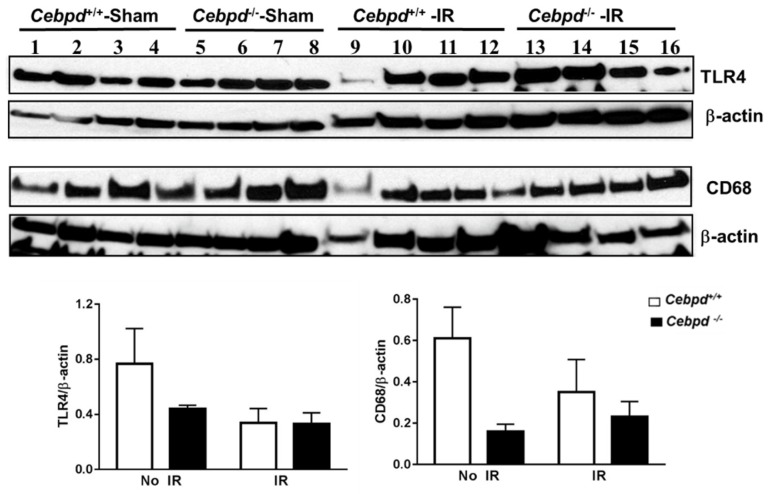
Expression of markers of inflammation and activated microglia in sham and irradiated *Cebpd*^+/+^ and *Cebpd*^−/−^ hippocampal extracts of aged mice. Immunoblotting of TLR4 and CD68 normalized to β-actin used as a loading control, *n* = 4 mice per genotype per treatment.

Exposure to IR is known to induce the expression of toll-like receptor 4 (TLR4) and pro-inflammatory cytokines which promote the increased recruitment of immune cells to clear the damaged tissue and/or dying cells. The activation of TLR4 is primarily in the microglia, so we also examined the expression of CD68, a marker of activated glia in the hippocampal extracts. We did not observe a significant difference in the expression of TLR4 nor CD68, which suggests that *Cebpd*-deficiency in aged mice did not further exacerbate IR-induced inflammation compared to unirradiated *Cebpd*^+/+^ mice (Figure 6).

**Figure 7:**
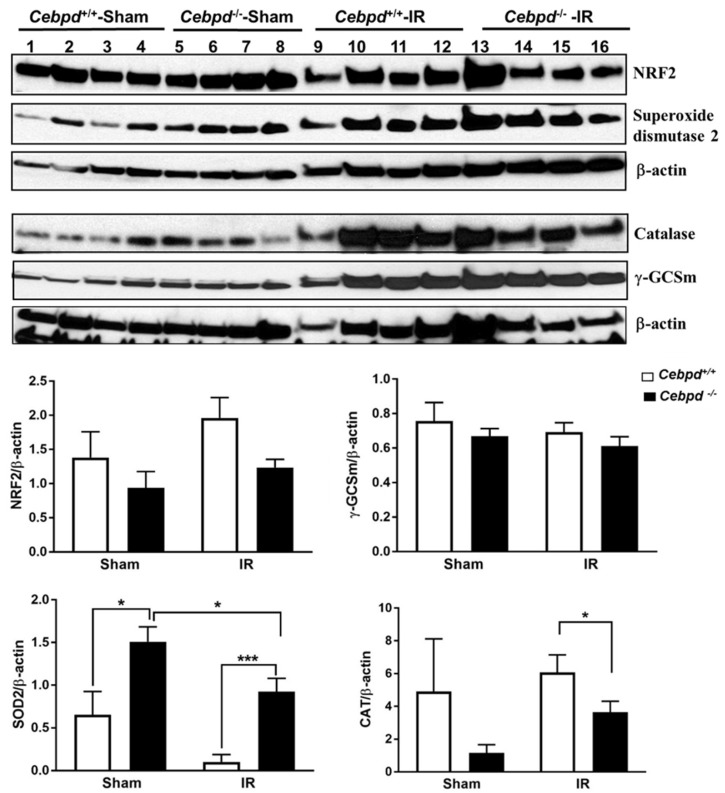
Expression of antioxidant response proteins in hippocampal extracts of sham and irradiated *Cebpd*^+/+^ and *Cebpd*^−/−^ mice. Immunoblotting of hippocampal extracts probed for NRF2, SOD2, CAT, γ-GCSm and β-actin, *n* = 4 mice per genotype per treatment. * *p* < 0.05; *** *p* < 0.001.

Exposure to IR induces increased oxidative stress and damage to cellular constituents and leads to cell death and damage to the tissues. The hippocampus, which is the center for neurogenesis, is sensitive to IR-induced oxidative stress which can be counteracted by the antioxidant response proteins such as nuclear factor (erythroid-derived 2)-like 2 (NRF2), superoxide dismutase 2 (SOD2), catalase (CAT), and gamma-glutamyl cysteine ligase subunit m (γ-GCSm). There was no significant difference between the genotypes in sham or irradiated groups in the expression of antioxidant response proteins such as NRF2 or γ-GCSm which is involved in the synthesis of the cellular antioxidant glutathione. The expression of SOD2 was significantly upregulated in *Cebpd*^−/−^-sham mice compared to *Cebpd*^+/+^-sham mice. Exposure to IR led to downregulation of the overall expression of SOD2 in *Cebpd*^+/+^ mice, however *Cebpd*^−/−^ mice still showed significantly higher expression (Figure 7). We also found that the post-IR hippocampal expression of CAT was significantly decreased in *Cebpd*^−/−^ mice compared to *Cebpd*^+/+^ mice (Figure 7). These results point to impairment in the oxidative stress response proteins and is suggestive of increased oxidative damage in the hippocampus which may play a role in neurocognitive deficits observed in *Cebpd*^−/−^ mice.

## 3. Discussion and Conclusions

Most of the clinical observations on radiation-induced neurocognitive impairments are based on the uncontrolled accidental exposure to radiation or the controlled cranial radiotherapy in cancer patients. Radiation exposure of the brain disrupts neurotransmission and elicits varying degrees of cognitive dysfunction [9,10,11]. While severe macroscopic tissue destruction and functional central nervous system (CNS) injury generally occur only after high radiation doses, lower doses do elicit moderate, acute changes [10]. Exposure to radiation gives rise to oxidative stress and neuroinflammation neurochemical mechanism detrimental to proper functionality of the CNS [40]. In young adult mice, significant reductions in proliferating and immature neurons are seen shortly after irradiation (i.e., 48 h) after irradiation [41]. In juvenile mice (age p21) 48 h after irradiation, the number of immature neurons is reduced 12% after 2 Gy to 75% after 10 Gy [42].

C/EBPδ expression is low to undetectable in most cell types and tissues. Activation of C/EBPδ has been observed in age-associated inflammatory diseases such as Alzhiemer’s disease and Parkinson’s disease [32,33]. Sterneck et al. previously demonstrated that *Cebpd* is expressed in distinct neuronal populations, including the granule neurons of the dentate gyrus and the pyramidal neurons of the hippocampus, and that young *Cebpd^−/−^* mice display an enhancement in contextual fear conditioning but not spatial learning in the Morris task [43]. Microarray analysis of genes expressed in the brains of young versus old mice revealed that the expression of *Cebpd* is not influenced by age [44].

The Y-maze is a simple 2-trial recognition test for measuring spatial recognition memory in animal experiments. The Y-maze test is based on the instinctive curiosity of rodents to explore novel areas without negative or positive reinforcements to the animals [45]. In the present study, one of the interesting findings was that aged *Cebpd*^+/+^ mice that received whole body radiation were not affected cognitively. However, *Cebpd*^−/−^-IR animals’ lack of curiosity about the novel arm, implying the inability to remember the start or the familiar arms, suggests deficits in the hippocampus-dependent process of short-term recall [46].

Recognition, a subtype of declarative memory, is composed of familiarity and recollection, which are processes dependent upon the hippocampus [38]. Recent findings are categorizing organized electrical activity in response to NOR within the hippocampus [23,38,47]. The dorsal hippocampus in particular is implicated in novel-object signaling. Within the dorsal hippocampus, the CA1 is paramount for object-novelty processing, as it is the main hippocampal output of the tri-synaptic pathway and broadcasts environmental novelty [48,49]. Our data showed that NOR was impaired significantly in *Cebpd*^−/−^-IR mice compared to *Cebpd*^−/−^-sham and *Cebpd*^+/+^-sham and *Cebpd*^+/+^-IR cohorts. However, since *Cebpd*^−/−^-IR mice explore objects equally during familiarization and exhibit no signs of neophobia, deficits in NOR are likely due to impaired learning and/or memory rather than reduced curiosity.

Dendritic branching alterations and spine morphology can disrupt synapse formation and/or stability, which ultimately can lead to neurological and cognitive disorders, such as autism spectrum disorders, Alzheimer’s disease, schizophrenia, anxiety, and depression [50]. Loss of dendritic arborization complexity would prohibit information processing and learning and memory formation that can manifest as cognitive dysfunction [51,52]. Neurons were once thought to be radioresistant cells because they do not divide, but we now know that they respond negatively to radiation. Our data showed significantly decreased dendritic length in the DG and CA1 regions of the hippocampus of irradiated *Cebpd*^−/−^ mice. In the DG, Sholl analysis of *Cebpd*^+/+^-sham mice compared with *Cebpd*^+/+^ -IR mice revealed significant reductions in dendritic length at 80–190 µm from the soma, with similar reductions at 90–190 µm in *Cebpd*^−/−^-sham mice when compared with *Cebpd*^−/−^-IR mice. Dendritic morphology has been implicated in the health of neurons [53]; these data suggest that C/EBPδ-deficiency enhanced neuronal damage after exposure to radiation. Our findings of changes in dendritic morphology are aligned with findings in the literature showing abnormal morphology and decreased complexity are associated with impaired learning and memory on behavioral testing [54].

It is known that cumulative oxidative stress and inflammation play a contributory role in the process of aging and are also associated with radiation injury [2,55,56,57]. In the present study, we did not find any significant changes in the expression of markers of inflammation such as TLR4 or in the expression levels of the marker for activated microglia, CD68. It is known that aging is also associated with chronic inflammation partly mediated by increased levels of damage-associated molecular patterns, which activate pattern recognition receptors of the innate immune system such as TLR4 [58]. It is perhaps possible that due to the baseline inflammation present in the aged mice, radiation does not further upregulate the expression of TLR4. Alternatively, it may be possible that the inflammatory peak is an early effect post-IR exposure and perhaps TLR4 may be upregulated at early time points in *Cebpd*^−/−^ mice, as observed in other tissues such as the intestine [59].

However, we found significant alterations in the post-irradiation expression of the antioxidant proteins, SOD2, and CAT between aged *Cebpd*^−/−^ and *Cebpd*^+/+^ mice It is known that the generation of ROS is considered the main cause of radiation-induced tissue injuries, and elevated levels of oxidative stress persist long after the initial irradiation [60]. We found significant upregulation of SOD2 in sham as well as irradiated *Cebpd^−/−^* mice. Interestingly, a study with proton irradiation reported that SOD2-deficient mice were protected from radiation-induced neurocognitive deficits compared to SOD2-wild type mice [61]. The hydrogen peroxide produced by SOD2 is further detoxified by the enzyme CAT which was found to be significantly downregulated in irradiated *Cebpd*^−/−^ mice. These findings are further supported by our previous studies with a transgenic mouse model overexpressing mitochondrial CAT which showed extended longevity [62] and significant protection of radiation-induced neurocoginitve deficits [63]. Further studies are needed to investigate the impaired expression of SOD2 and CAT in the specific neuronal cells of the hippocampus by immunostaining and whether SOD2 knockdown or CAT overexpression can alleviate the post-irradiation loss of cognitive functions in *Cebpd^−/−^* mice.

Taken together, our results show that *Cebpd*-deficiency promotes radiation-induced deficits in short-term memory and spatial learning in aged mice that may be due to an impaired ability to detoxify IR-induced oxidative stress.

## 4. Materials and Methods

### 4.1. Ethics Statement

This study was carried out in strict accordance with the recommendations in the Guide for the Care and Use of Laboratory Animals of the National Institutes of Health and approved by the Institutional Animal Care and Use Committee of the University of Arkansas for Medical Sciences, animal use protocol number #3511, approved on 5/20/2014).

### 4.2. Animals

*Cebpd*-heterozygous breeder mice were backcrossed for more than 20 generations to the C57BL/6 strain background. Genotyping was done as described previously [34]. In all the studies, 15-month-old male *Cebpd*^+/+^ and *Cebpd*^−/−^ littermate mice were used. The animals were housed in the Division of Laboratory Medicine (DLAM, University of Arkansas for Medical Sciences, Little Rock, AR, USA) under standardized conditions with controlled temperature and humidity and a 12-h day, 12-h night light cycle. Brain tissues were harvested from sham mice and from irradiated mice at day 11 post-IR following isoflurane inhalation to minimize suffering and the animals were euthanized by cervical dislocation.

### 4.3. Irradiation of Mice

*Cebpd*^+/+^ and *Cebpd^−/−^* mice were exposed to TBI administered in a Mark I irradiator (J. L. Shepherd & Associates, San Fernando, CA, USA). Dose uniformity was assessed by an independent company (Ashland Specialty Ingredients, Wilmington, DE, USA) with radiographic film and alanine tablets. Alanine tablets were analyzed by the National Institute of Standards and Technology (Gaithersburg, MD, USA) and demonstrated a dose rate of 1.14 Gy/min at 21 cm from the source. For each experiment, the dose rate was corrected for decay.

The total dose of TBI used in the present study was 8.5 Gy. We have previously reported that 3-month-old *Cebpd*^+/+^ mice exposed to 8.5 Gy led to 100% mortality by days 9–13 post-TBI compared to *Cebpd*^+/+^ mice, which showed 40% mortality by days 11–13 post-TBI [34]. Aged (15 months old) *Cebpd^−/−^* mice display about 55% mortality by days 8–12 compared to 12.5% mortality at day 15 post-TBI dose of 8.5 Gy (Pawar et al., unpublished results, data not shown). Hence, we chose the timepoint of 7–10 days post-irradiation for the behavior studies followed by tissue harvest on day 11 post-irradiation to examine the morphological changes and molecular changes in the hippocampus.

### 4.4. Behavioral Methods

In the sham group, *n* = 5 mice for each genotype, where in the IR group, *Cebpd*^+/+^ mice (*n* = 7) and *Cebpd*^−/−^ mice (*n* = 5) were used for the behavior studies. The figure below depicts the timepoints for the behavior studies that were conducted prior to tissue harvest.

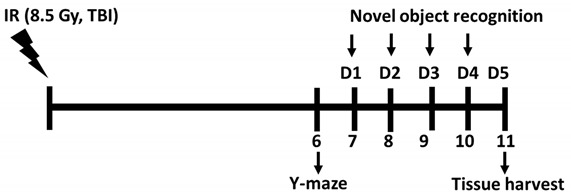



#### 4.4.1. Y-Maze

At day 6 post-irradiation, *Cebpd*^+/+^ and *Cebpd*^−/−^ mice were first tested in the Y-maze, which did not rely on either negative or positive reinforcement. The maze was constructed out of acrylic and consisted of three similar arms (45L x 7W x 14H cm): a “start” arm where animals were placed initially, a “familiar” arm, and a “novel” arm. The familiar and novel arms each contained an object of different size and shape mounted at the end of the arm. Animals were placed in the start arm facing away from the center of the maze. The familiarization session consisted of free exploration of the start and familiar arms for 10 min. Four hours later, the testing session was held; animals were again placed in the maze, this time with access to all arms. Allocation of arms (start, familiar, or novel) was counterbalanced between each experimental group. Trials lasted for 10 min, and center- and nose-points were recorded throughout each session. An arm entry was counted when all four limbs of the mouse entered an arm. All experimental arenas were wiped clean with 20% ethanol after each trial. All behavioral experiments were conducted during the light cycle under dimly-lit (white light) conditions, after a minimum of one hour of acclimation. Behavioral experiments were recorded on a charge-coupled device video camera, located above the maze for automatic behavioral analysis with EthoVision XT software version 11 (Noldus Information Technology, Leesburg, VA, USA) as described previously [23].

#### 4.4.2. Novel Object Recognition

On day 7 post-irradiation, *Cebpd^+/+^* and *Cebpd^−/−^* mice were tested for novel object recognition (NOR) with a 4-day procedure in which animals freely explored an arena for 10 min each day. The arena was a cube consisting of an aluminum floor, acrylic walls (41L × 41W × 35H cm), and an open ceiling. The first two days (days 7 and 8 post-TBI) served as habituation learning days, in which mice were able to explore the empty arena (effectively serving as open field tests); locomotor activity was measured at this stage. The familiarization phase occurred on day 3 (day 9 post-TBI), when animals explored an arena containing two identical objects (cell-culture flasks filled with sand). Novel object recognition testing occurred on day 4 (day 10 post-TBI); here, a now-familiar object was replaced with a novel object (large LEGO^®^ blocks assembled to the size of the cell-culture flasks) [64]. Animals were placed in the center of the arena parallel to the objects to avoid bias. NOR testing relies on the animals’ natural inclination to explore novel objects in their environment (untreated animals should spend significantly more time exploring the novel object). The tracking software was programmed to track animal center-points for the habituation trials and nose-points during familiarization and testing trials.

#### 4.4.3. Golgi Staining

Shortly after behavioral testing, animals were euthanized, and their brains were collected at day 11 post-8.5 Gy and dissected along the midsagittal plane and half of the hippocampus was harvested for Golgi staining. The Golgi method of staining has long proven to be a reliable method for assessing dendrite and dendritic spine dynamics due to various treatments, because of its resistance to fading or photobleaching over time [65,66]. We adapted a staining protocol and used the reagents contained in the superGolgi kit (Bioenno Tech, Santa Ana, CA, USA) [67]. Right hemispheres were immediately impregnated in a potassium dichromate solution for two weeks (*n* = 5). Next, sections were immersed for at least 48 h in a post-impregnation buffer. Samples were sectioned at 200 µm in 1× PBS along the coronal plane. Samples were then transferred into wells and washed with 0.01 M PBS buffer (pH 7.4) with Triton X-100 (0.3%) (PBS-T). Immediately after washing, samples were stained with ammonium hydroxide and then immersed in a post-staining buffer. Sections were again washed in PBS-T, mounted on 1% gelatin-coated slides, and allowed to dry. Sections were finally dehydrated with ethanol solutions, followed by cleaning in xylene, and coverslipped with Permount^TM^ (Thermo Fisher Scientific, Waltham, MA, USA).

#### 4.4.4. Dendritic Morphology Quantification

All dendritic morphology data were collected blinded with regard to experimental conditions on *n* = 5 mice per genotype per treatment group. We performed quantification of morphological characteristics of the granular and pyramidal neurons contained in the hippocampal formation using techniques that included Sholl analyses (Figure S2, Supplementary Materials), total dendritic length, number of branch points, and dendritic complexity index (DCI). Multiple Z-stack images of neurons were collected with the aid of a computer-assisted neuron tracing using the Neuroexplorer component of the Neurolucida program (Ver. 11, Microbrightfield, Inc., Williston, VT, USA). Sholl analysis was used to assess the amount and distribution of the arbor at increasing radial distances from the cell body [68]. Radii were set to extend in 10 µm intervals from the soma. The length of each dendritic branch, within each progressively larger circle, was counted from the soma, with respect to three dimensions. This provided information about the amount and distribution of individual dendrites.

We then performed branch-point analyses. Branch points occur at bifurcations of the dendrite when a branch divides into two sub-branches. Branch-point analysis depends on the number of bifurcations and the order of the points [69]. Lower branch-point orders represent proximal regions of the tree, whereas larger branch-point orders characterize distal regions. We used the branch-point analysis to determine the complexity of dendritic arborization, because the complexity of the dendritic tree is an important phenotypic component of branching analysis. DCI was determined by the following equation: DCI=∑ (branch tip orders + # of branch tips) × (total dendritic length/total number of primary dendrites). In the CA1 apical and basal regions, dendrites were analyzed separately.

#### 4.4.5. Immunoblotting of Hippocampal Extracts

Hippocampal tissues were harvested from sham and irradiated *Cebpd*^+/+^ and *Cebpd*^−/−^ mice at day 11 post-irradiation and protein extracts were prepared using an IBI Scientific DNA/RNA/Protein Extraction kit (MIDSCI, St. Louis, MO, USA), and the protein was quantified using a Nanodrop 2000c A280 (Thermo Scientific, Waltham, MA, USA). The protein samples were mixed with 2× sodium dodecyl sulfate polyacrylamide gel electrophoresis sample buffer and boiled for 5 min. A 35µg portion of total protein per sample was separated by a 4–20% gradient sodium dodecyl sulfate polyacrylamide gel electrophoresis, electrotransferred to polyvinylidene fluoride (PVDF) filters at 40 V and 4 °C for 120 min, blocked with 5% non-fat milk at room temperature for 1 h, and incubated with primary antibodies specific to NRF2 (sc-722), SOD2 (sc-30080), CAT (sc-50508), γ-GCSm (sc-55586), TLR4 (sc-293072), and CD68 (sc-59103) (Santa Cruz Biotechnology, Dallas, TX, USA), and β-actin (4790, Cell Signaling Technology, Danvers, MA, USA) monoclonal antibodies overnight at 4 °C. The membranes were washed three times with Tris-buffered saline/Tween-20 (TBST), incubated with secondary antibody for 60 min, washed three times with TBST, and visualized by enhanced chemiluminiscence. β-actin expression was used as the internal reference. The band intensities were measured by densitometry using NIH ImageJ analysis.

#### 4.4.6. Statistical Analyses

We expressed data as a mean ± the standard error of the mean (SEM). We analyzed the behavioral data throughout the 10-minute length of each test. Behavioral assays comparing visits or time spent in apparatus areas by individual treatment groups were analyzed via ANOVA. NOR discrimination ratio (DR) was calculated by the following formula: (*NOR*) DR = (novel object visits - familiar object visits)/(novel object visits + familiar object visits). For measures of dendritic length, two-way repeated-measures ANOVA was conducted for the effects of radiation (between-subjects variable) and distance from the cell soma (Sholl radius, repeated-measures variable); Holm’s correction to control for multiple comparisons post-hoc tests followed, when appropriate. Densitometry data were analyzed by unpaired Student’s *t*-test. All statistical analyses were conducted with GraphPad Prism 7.0 software (La Jolla, CA, USA) in a 95% confidence interval, and *p* < 0.05 was considered significant.

## Figures and Tables

**Table 1:** Analysis of dendritic morphology of dentate gyrus (DG) granule neurons in aged *Cebpd*^+/+^ and *Cebpd*^−/−^ mice. **** Bold figures represent significant compared to *Cebpd*^−/−^-IR.

Cell Type and Measurements	*Cebpd*^+/+^-Sham (mean ± SEM)	*Cebpd*^−/−^-Sham (mean ± SEM)	*Cebpd*^+/+^-IR (mean ± SEM)	*Cebpd*^−/−^-IR (mean ± SEM)
DG				
Total Dendritic Length	1224 ± 77.77	1313 ± 86.06	987.4 ± 144.5	587.7 ± 36.45
Total Number of Branch Points	8.92 ± 0.57	8.08 ± 0.57	5.8 ± 0.72	3.8 ± 0.21
Complexity	30796 ± 6401	38947 ± 3424	15985 ± 5436	7050 ± 1350

**Table 2:** Analysis of CA1 apical and basal neurons in aged *Cebpd*^+/+^ and *Cebpd*^−/−^ mice. **** Bold figures represent significant compared to *Cebpd*^−/−^-IR.

Cell Type and Measurements	*Cebpd*^+/+^-Sham (mean ± SEM)	*Cebpd*^−/−^-Sham (mean ± SEM)	*Cebpd*^+/+^-IR (mean ± SEM)	*Cebpd*^−/−^-IR (mean ± SEM)
**CA1 Apical**				
**Total Dendritic Length**	**839.8 ± 82.52**	**677.6 ± 74.02**	519.1 ± 43.86	414.6 ± 32.46
**Total Number of Branch Points**	**8.00 ± 0.96**	**7.13 ± 0.58**	5.3 ± 0.39	4.05 ± 0.45
**Complexity**	**43356 ± 9937**	**29426 ± 6042**	17154 ± 1491	8699 ± 1649
**CA1 Basal Measurements**				
**Total Dendritic Length**	**1301 ± 173.34**	**823.01 + 52.91**	**788.8 ± 22.93**	472.6 ± 28.61
**Total Number of Branch Points**	**9.90 ± 1.35**	**7.00 + 0.64**	**6.95 ± 0.49**	3.85 ± 0.52
**Complexity**	**20722 ± 5219**	8366 + 1556	9275 + 1671	3749 ± 540.5

## Supplementary Materials

The following are available online at http://www.mdpi.com/1422-0067/20/4/885/s1, Figure S1: Discrimination ratio of sham and irradiated aged *Cebpd*^+/+^ and *Cebpd*^−/−^ mice. Figure S2: Combined Sholl analyses of neurons in DG and CA1 apical and CA1 basal regions depicted in Figure 3, Figure 4 and Figure 5. Figure S3: Representative tracings of DG granule neurons superimposed over concentric rings (10 µM) used for Sholl analysis.
