# Supplementary material for: Loss of C/EBPδ Exacerbates Radiation-Induced Cognitive Decline in Aged Mice due to Impaired Oxidative Stress Response
Source: Int J Mol Sci. 2019 Feb 18;20(4):885. doi: 10.3390/ijms20040885 (PMC6412914; doi:10.3390/ijms20040885)
Supplement: Supplementary file 1 [file ijms-20-00885-s001.pdf]

Supplementary Materials

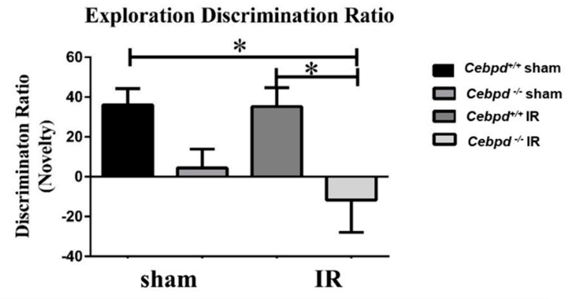

Supplementary Figure 1. Discrimination ratio of sham and irradiated aged *Cebpd*$^{+/+}$ and *Cebpd*$^{-/-}$ mice. The DI shows reduced exploratory preference for the novel object over the familiar object in KO-IR resulting in negative discrimination ratio. *N*=5-7 mice/treatment. DI, discrimination index. Average ± SEM; *p < 0.05.

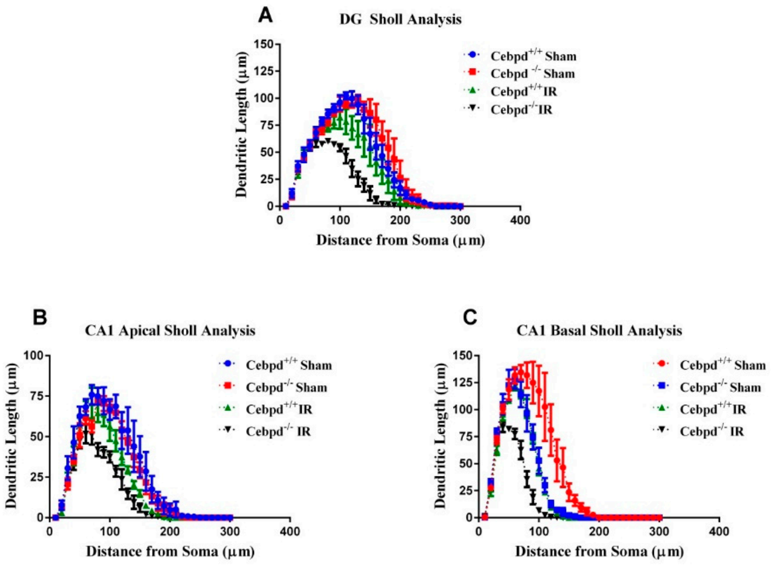

Supplementary Figure 2. Combined Sholl analyses of neurons in DG, CA1 apical and CA1 basal regions depicted in Figures 3, 4 and 5.

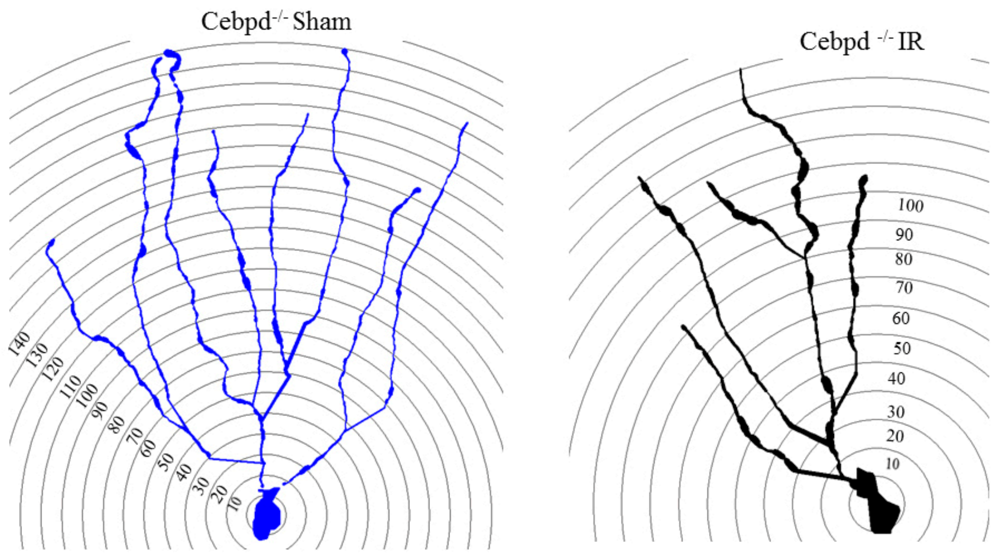

Supplementary Figure 3: Representative tracings of DG granule neurons superimposed over concentric rings (10 μM) used for Sholl analysis.
